# A two-stage forecasting model using random forest subset-based feature selection and BiGRU with attention mechanism: Application to stock indices

**DOI:** 10.1371/journal.pone.0323015

**Published:** 2025-05-09

**Authors:** Shafiqah Azman, Dharini Pathmanathan, Vimala Balakrishnan

**Affiliations:** 1 Institute of Mathematical Sciences, Faculty of Science, Universiti Malaya, Kuala Lumpur, Malaysia; 2 Universiti Malaya Centre for Data Analytics, Universiti Malaya, Kuala Lumpur, Malaysia; 3 Center of Research for Statistical Modelling and Methodology, Faculty of Science, Universiti Malaya, Kuala Lumpur, Malaysia; 4 Department of Information Systems, Faculty of Computer Science and Information Technology, Universiti Malaya, Kuala Lumpur, Malaysia; 5 College of Informatics, Korea University, Seoul, Republic of Korea.; Federal University of Petroleum Resources Effurun, NIGERIA

## Abstract

The heteroscedastic and volatile characteristics of stock price data have attracted the interest of researchers from various disciplines, particularly in the realm of price forecasting. The stock market’s non-stationary and volatile nature, driven by complex interrelationships among financial assets, economic developments, and market participants, poses significant challenges for accurate forecasting. This research aims to develop a robust forecasting model to improve the accuracy and reliability of stock price predictions using machine learning. A two-stage forecasting model is introduced. First, a random forest subset-based (RFS) feature selection with repeated k-fold cross-validation selects the best subset of features from eight predictors: highest price, lowest price, closing price, volume, change, price change ratio, and amplitude. These features are then used as input in a bidirectional gated recurrent unit with an attention mechanism (BiGRU-AM) model to forecast daily opening prices of ten stock indices. The proposed model exhibits superior forecasting performance across ten stock indices when compared to twelve benchmarks, evaluated using root mean squared error (RMSE), mean absolute error (MAE), and the coefficient of determination, R2. The improved prediction accuracy enables financial professionals to make more reliable investment decisions, reducing risks and increasing profits.

## 1. Introduction

Analyzing stock market data is challenging due to non-stationarity, dynamic volatility, and the complex interrelationships between financial assets, economic developments, and market participants, which contribute to significant noise [1,2]. Reliable prediction of stock indices assists investors in maximizing profit while minimizing risk. Researchers often encounter difficulties when using traditional statistical models for forecasting due to the complexity, nonlinearity, and non-stationarity of stock market data [3]. The main challenge in predicting these kinds of data lies in effectively identifying the important features from a wide range of possible predictors, including historical prices, trading volumes, macroeconomic indicators, and market sentiment data. Without proper feature selection, forecasting models may suffer from high dimensionality, leading to overfitting, increased computational complexity, and reduced predictive accuracy [4].

### 1.1. Statistical time series models

Statistical time series models such as the autoregressive integrated moving average (ARIMA) model [5] and the generalized autoregressive conditional heteroscedasticity (GARCH) model [6] as an extension of Engle’s autoregressive conditional heteroscedasticity (ARCH) model [7], serve as important models in the analysis and forecasting of financial data. ARIMA models are designed to analyze and predict future points in a series by capturing trends, seasonality, and cyclical patterns through a combination of autoregressive, integrated, and moving average components. This makes ARIMA suitable for univariate time series data where past values and errors help predict future values. On the other hand, GARCH models specialize in modeling and forecasting volatility, addressing the heteroscedasticity present in financial time series data. By accounting for volatility clustering, where periods of high volatility tend to cluster together, GARCH models provide a robust framework for understanding and predicting the variability in stock prices. To enhance the capability of these models, hybrid statistical models with deep learning were introduced [8,9].

However, these methods are often not robust enough for dynamic financial markets due to the presence of significant noise caused by factors like economic conditions, political conflicts, large investors’ monopolization, and unpredictable trading patterns [10]. This highlights the need for advanced models capable of capturing the evolving characteristics of financial data.

### 1.2. Random forest for feature selection

To improve forecasting accuracy, effective feature selection is crucial. By selecting the important features and removing redundant ones, overfitting can be prevented, thus enhancing the efficiency of the forecasting model [4]. Random forest (RF) [11] is a powerful machine learning method for classification and regression problems. It is an ensemble-based algorithm constructed from decision tree predictors. The RF method leverages the law of large numbers, thereby effectively mitigating the issue of overfitting. In a regression context for feature selection, RF predictions equate to the average of predictions made by all individual decision trees. The advantage of RF for regression-based feature selection lies in its capability to effectively reduce computational complexity while capturing and modeling nonlinear and complex dependencies among features, thereby improving overall predictive performance [12,13].

In the study of feature selection for stock price prediction, RF is more commonly used as a classification model, and there is limited research on its use as a regression model. In this review, only studies where the RF regression model is employed are considered. RF with leave-one-out cross-validation was used in [14] to select 42 microeconomic variables before predicting stock prices with the LSTM model. In their study, they found that using RF for feature selection improved the model’s predictive accuracy, as it focuses on the most relevant variables, thus enhancing the overall performance of the LSTM model. RF is also used as a feature selection technique in [15] for predicting the Chinese stock market. In terms of feature selection based on importance, these studies are similar. They found that RF effectively identified key predictors from a vast dataset, which improved the accuracy of their stock market predictions. A more recent review study [16] emphasized RF’s effectiveness in handling large and noisy financial datasets, proving its importance in feature selection and predictive analysis for stock market data.

### 1.3. Deep learning forecasting models

Deep learning models are increasingly used for forecasting financial time series data due to their ability to address the shortcomings of traditional statistical models. Recurrent neural networks (RNNs) are particularly useful for capturing dynamic temporal features in stock market data, aiding in self-learning and sequential data analysis [17]. However, RNNs suffer from the vanishing gradient problem with long-sequenced data. The vanishing gradient problem in gradient-based training occurs when the gradients of network weights become extremely small and eventually approach zero, making it challenging for the model to update its weights effectively [18].

To address this, long-short-term memory (LSTM) model [19] and gated recurrent unit (GRU) model [20] were developed. Numerous forecasting models based on LSTM and GRU architectures have been proposed, including the model incorporating LSTM with convolutional neural network (CNN) [21] and the hybrid LSTM-GRU model [22]. A review study [23] emphasized LSTM’s preference among researchers for financial time series forecasting, however, a more recent studies [24,25] found that GRU outperforms LSTM in terms of overall performance, particularly with smaller datasets.

### 1.4. Bidirectional Gated Recurrent Unit (BiGRU), Attention Mechanism (AM) and BiGRU-AM

Bidirectional GRU models have shown significant potential in forecasting financial data by effectively utilizing past and future information to improve prediction accuracy and prevents information loss [26,27]. It allows the model to effectively model temporal dependencies throughout the sequence and in turn outperform its unidirectional GRU counterpart by ensuring a more comprehensive understanding of the temporal relationships within the data [28]. Recent works that successfully incorporated BiGRU to forecast stock price indices include the models by [29,30].

The attention mechanism (AM) is modelled after how the human brain selectively focuses on essential information. Its underlying concept is to prioritize essential data while minimizing less important aspects. AM improves processing efficiency by dynamically altering focus to increase sensitivity to critical inputs [31]. The implementation of AM in deep learning was first proposed by [32] for language translation. AM assigns higher weights to specific input components before mapping them to the final output, and has since become widely used in Natural Language Processing (NLP). The implementation of AM for time series data was first introduced by [33], assigning importance to certain input features from the past and future, allowing different attention weights to critical temporal features at different time steps. AM has since been widely used in deep learning models for predicting time series data [34, 35, 36].

The BiGRU-AM hybrid model has been successfully applied to the classification of HTTPS traffic [37], human emotions from EEG signals [38], and air target tactical intention recognition [39]. However, it has never been used in the setting of regression problems nor on financial time series data.

### 1.5. Contributions of the study

This study proposes a two-stage forecasting model for financial data with the following key contributions:

- A random forest subset-based feature selection method is developed, combined with repeated k-fold cross-validation to identify the best subset of features from eight potential predictor features.
- The implementation of BiGRU-AM model in a regression setting is introduced for the first time, specifically for predicting stock prices.
- A comprehensive comparative study was conducted to evaluate the performance of the proposed two-stage forecasting model against twelve benchmark models, demonstrating the effectiveness of the proposed approach in predicting the daily opening prices of ten stock indices from January 2000 to February 2022.

The remainder of this paper is organized as follows: Section 2 describes the data used and introduces the architecture of the model proposed; Section 3 reviews the results and discussion; and Section 4 concludes.

## 2. Methodology

### 2.1. Data description

To evaluate the performance of the proposed model, ten major stock indices were chosen [40], which are Dow Jones, Nasdaq, Nikkei 225, FTSE 100, S&P 500, CAC 40, IPC, DAX, AEX, and BEL 20. The datasets range from January 2000 to February 2022 and are made up of consecutive trading days (excluding weekends and public holidays). Table 1 shows the country, period (depending on data availability), and length of each dataset. The historical data for the stock indices is retrieved from Yahoo! Finance (accessed on 31 March 2022).

**Table 1:** Country-specific stock indices and periods observed for model evaluation.

Stock Indices	Country	Period Observed	Length of Data (Consecutive Days)
Dow Jones	United States	3/1/2000–25/2/2022	5574
Nasdaq	United States	3/1/2000–25/2/2022	5574
Nikkei 225	Japan	4/1/2000–25/2/2022	5427
FTSE 100	United Kingdom	4/1/2000–25/2/2022	5598
S&P 500	United States	3/1/2000–25/2/2022	5574
CAC 40	France	3/1/2000–25/2/2022	5662
IPC	Mexico	3/1/2000–25/2/2022	5560
DAX	Germany	3/1/2000–25/2/2022	5622
AEX	Netherlands	3/1/2000–25/2/2022	5665
BEL 20	Belgium	3/1/2000–25/2/2022	5659

Table 2 lists the eight features chosen from [41] for inclusion in this study’s multivariate analysis. The opening price (F1), highest price (F2), lowest price (F3), closing price (F4), and volume (F5) can all be imported directly from Yahoo! Finance, while the change (F6), price change ratio (F7), and amplitude (F8) can be calculated as follows:

**Table 2:** Features investigated for multivariate forecasting.

Features	Description
F1	Opening price
F2	Highest price
F3	Lowest price
F4	Closing price
F5 (unit: 10^8^)	Volume
F6	Change
F7 (unit:%)	Price change ratio
F8 (unit:%)	Amplitude


Change=Closing  price  of  the  day−Closing  price  of  the  previous  day,

$$\text{Price }\;\;\;\;\text{ change }\;\;\;\;\text{ ratio}=\frac{\text{Change }\;\;\;\;\left(\text{F}5\right)}{\text{Closing }\;\;\;\;\text{ price }\;\;\;\;\text{ of }\;\;\;\;\text{ the }\;\;\;\;\text{ previous }\;\;\;\;\text{ day}},$$

$$\text{Amplitude}=\frac{\left|\text{Highest }\;\;\;\;\text{ price}-\text{Lowest }\;\;\;\;\text{ price}\right|}{\text{Closing }\;\;\;\;\text{ price }\;\;\;\;\text{ of }\;\;\;\;\text{ the }\;\;\;\;\text{ previous }\;\;\;\;\text{ day}}.$$

The stock indices data are divided into 3 sets, where the first 80% is the training set, the following 10% is the validation set, and the remaining 10% is the test set.

### 2.2. Stage 1: Random Forest Subset-based (RFS) for Feature Selection

In the first stage, the Random Forest subset-based (RFS) feature selection method identifies the best subset of features for predicting stock indices prices by generating all possible feature subsets, using the RF regression model to evaluate each subset’s prediction accuracy through repeated k-fold cross-validation, and selecting the subset with the lowest Mean Absolute Error (MAE).

#### 2.2.1. Random Forest (RF) for regression.

**Fig 1:**
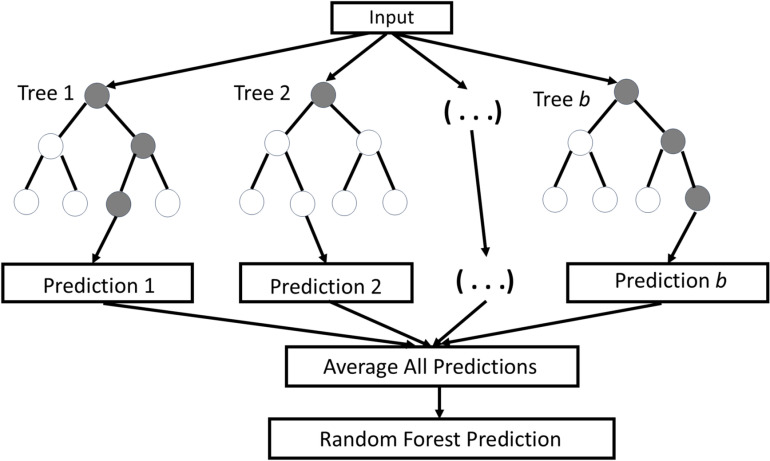
Random Forest regression model with b decision trees.

For RF in regression setting, the input vector x consists of independent variables, and the output Y is numerical. The training data $\left(x_{1},y_{1}\right),\ldots ,\left(x_{t},y_{t}\right)$ is drawn from the joint distribution of (X,Y). An example of RF regression model with n decision trees is shown in Fig 1.

The process for RF for regression is

For b=1 to B (number of trees in the forest):

1. A bootstrap sample $Z_{b}$ was drawn from the training data.
2. A decision tree $T_{b}$ was trained on $Z_{b}$.
3. At each node, the best split from a random subset of m features were selected.
4. The final prediction for a new input x was the average of predictions from all individual trees: $\hat{y}=\frac{1}{B}\sum {\mathrm{\backslash nolimits}}_{b=1}^{B}T_{b}\left(x\right)$.

Detailed derivations and proofs can be found in [11].

#### 2.2.2. Random Forest Subset-based (RFS) with Repeated k-fold Cross-Validation.

The procedure for the RFS feature selection is as follows:

1. **Generating Feature Subsets**: Create all possible subsets ${\text{S}}_{\text{j}}=\left\{{\text{F}}_{1},\ldots ,{\text{F}}_{m}\right\}$, of the input features ${\text{F}}_{\text{e}}\;\;\;\;=\;\;\;\;\left\{{\text{F}}_{1},\;\;\;\;{\text{F}}_{2},\;\;\;\;\ldots ,\;\;\;\;{\text{F}}_{\text{m}}\right\}$, such that i $=2^{m}-1,$ where m=no. of  features. In this study, the input features ${\text{F}}_{\text{e}}\;\;\;\;$are features{F2, F3, F4, F5, F6, F7, F8} from Section 3.1, while feature F1 is the output feature.
2. **Repeated** k**-Fold Cross-Validation**: Divide data to k-fold and use k−1 of the folds as training data. Evaluate subset of features in each fold and repeat for N multiple times to ensure reliable performance.
3. **Selecting the Optimal Subset**: Choose the subset of features with the lowest average MAE.

The RFS feature selection technique does an exhaustive search, thereby selecting the best subset of features from all possible combination of features and hence reducing likelihood of overfitting. Repeating the k-fold cross-validation N times mitigates the issue of a model being favored due to specific data partitioning, ensuring more reliable performance estimates [42]. This methodology offers a reliable and more robust feature selection that is essential in enhancing the prediction accuracy of the BiGRU-AM model.

**Fig 2:**
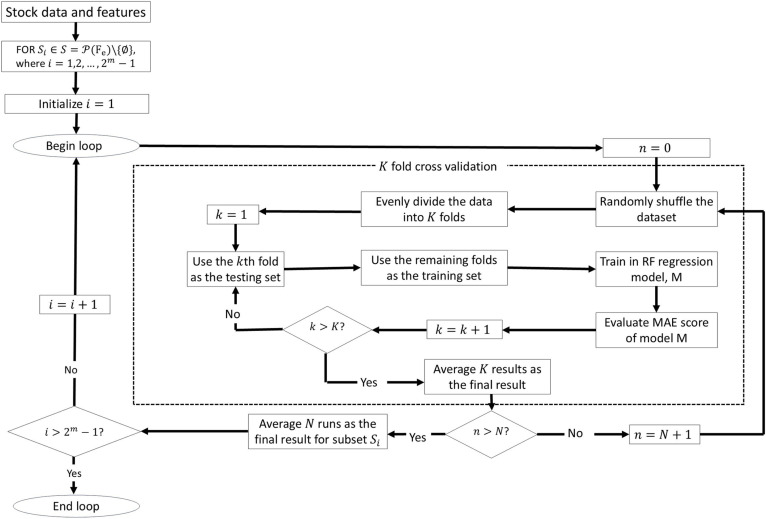
Flowchart of RFS feature selection with K folds and N repeats.

A more detailed flowchart of the procedure is shown in Fig 2 and the pseudocode algorithm for the proposed RFS feature selection is provided as Algorithm 1.

**Table:** 

Algorithm 1 Pseudo code for RFS feature selection method
**Inputs:**
Set of n original features ${\text{F}}_{\text{e}}=\left\{{\text{F}}_{1},\ldots ,{\text{F}}_{m}\right\}$
Data frame $X_{train}$ with row=observations and $\text{column}=\text{features }\;\;\;\;{\text{F}}_{\text{e}}$ and $y_{train}$
**Function:** {Enumerate all possible subsets of ${\text{F}}_{\text{e}}$ and perform subset-based feature selection}
**for** each subset ${\text{S}}_{\text{j}}=\left\{{\text{F}}_{1},\ldots ,{\text{F}}_{m}\right\}$ where $𝒫\left({\text{F}}_{\text{e}}\right)$ is the power set of ${\text{F}}_{\text{e}}$ **do**
Fit input $X_{train}\left[,i\right]$ and output $y_{train}$ on RF regressor model, M
Perform repeated k -fold CV on model M with 10 folds and 5 repeats
Evaluate CV score by using MAE for each repeat
Take the mean of CV score
**end for**
**Output:** Best subset of features with lowest CV error score

### 2.3. Stage 2: The Bidirectional GRU with Attention Mechanism (BiGRU-AM) Model for Stock Price Forecasting

In the second stage, implementation of the BiGRU-AM model in regression setting is introduced for stock price forecasting using the best subset of features selected in the first stage.

#### 2.3.1. Gated Recurrent Unit (GRU) and Bidirectional GRU.

The GRU’s model structure is made up of two gates: the update gate and the reset gate. The update gate determines the importance of previous time step information and controls the amount of information received that must be passed to the future. The reset gate, on the other hand, governs how much past information is erased [20].

The computation for the update gate, $z_{t}$ and the reset gate, $r_{t}$ are as follows:


$$\begin{array}{c} z_{t}=\sigma \left(x_{t}{\mathrm{\backslash bold}W}_{xz}+h_{t-1}{\mathrm{\backslash bold}W}_{hz}+b_{z}\right), \end{array}$$

$$\begin{array}{c} r_{t}=\sigma \left(x_{t}{\mathrm{\backslash bold}W}_{xr}+h_{t-1}{\mathrm{\backslash bold}W}_{hr}+b_{r}\right), \end{array}$$

$$\begin{array}{c} {\tilde{h}}_{t}=\sigma (x_{t}*{\mathrm{\backslash bold}W}_{xh}+\left(r_{t}*h_{t-1}\right){\mathrm{\backslash bold}W}_{hh}+b_{h)}, \end{array}$$

$$h_{t}=z_{t}*{\tilde{h}}_{t}+\left(1-z_{t}\right)h_{t-1},$$

where $x_{t}$ is the new input, $h_{t-1}$ is the previous memory, $h_{t}$ is the current memory and is the candidate hidden state for t=1,2,…,n. $\begin{array}{c} {\mathrm{\backslash bold}W}_{xz} \end{array}$, $\begin{array}{c} {\mathrm{\backslash bold}W}_{hz} \end{array}$, $\begin{array}{c} {\mathrm{\backslash bold}W}_{xr} \end{array}$, $\begin{array}{c} {\mathrm{\backslash bold}W}_{hr} \end{array}$, $\begin{array}{c} {\mathrm{\backslash bold}W}_{xh} \end{array}$, $\begin{array}{c} {\mathrm{\backslash bold}W}_{hh} \end{array}$ are the weight matrices and $b_{z}$, $b_{r}$, $b_{h}$ are the bias vectors. * denotes the Hadamard product between two matrices and σ denotes the activation function.

A bidirectional structure of GRU, specifically named Bidirectional GRU (BiGRU) processes data in two directions: forward and backward. This dual processing enables the model to fully extract relevant information from both the front and back of the sequence data, enhancing the prediction performance by considering the entire context of the data. This architecture enables the model to capture complex temporal dependencies by considering the entire sequence during training [43]. However, while the model is trained on complete sequences, during actual forecast of the time series data, the model utilizes only past data up to the current time point. The backward processing during training serves to enhance the learning of temporal patterns, leading to improved predictive performance.

#### 2.3.2 Attention Mechanism (AM).

As introduced by [33], the weighted feature ${\hat{x}}_{t}$replaces the original input $x_{t}$as the input of the neural network for time series data. Specifically, the implementation of AM can be computed as follows:


$$\begin{array}{c} e_{t}=Attention\left(x_{t},s_{t-1},\alpha_{t-1}\right), \end{array}$$

$$\alpha_{tj}=\frac{\text{exp}\left(e_{tj}\right)}{\sum_{j=1}^{n}exp\left(e_{tj}\right)},$$

$${\hat{x}}_{tj}=\alpha_{tj}x_{tj},$$

Where the attention score $e_{t}$ is the attention score is computed based on the input $x_{t}$, previous state $s_{t-1}$ and previous attention weight $\alpha_{t-1}$ for time t=1,…,T and observation j=1,…,n. Details on how the mechanism of AM works can be found in [33].

### 2.4 Overall Model Architecture

**Fig 3:**
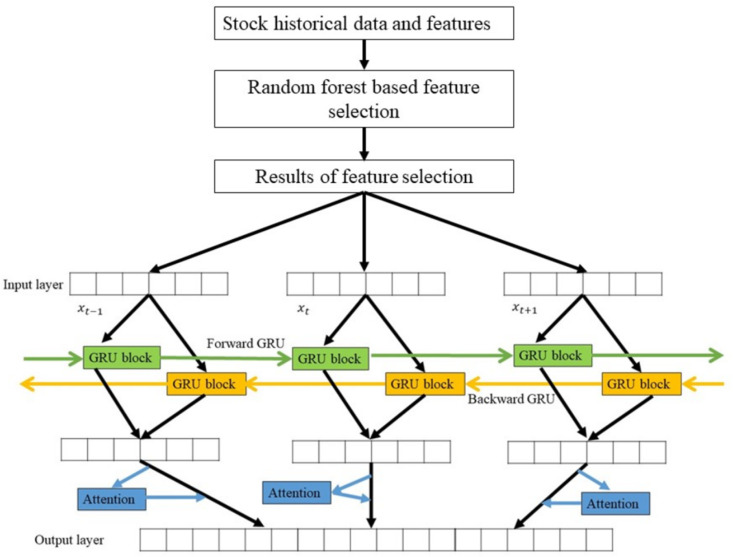
Proposed model framework flowchart.

The overall flow of the proposed model, which combines a first stage of RFS feature selection and a second stage of BiGRU-AM model forecasting, is shown in Fig 3. In the first stage, the features (F2–F8) are processed as the input variables to the RFS feature selection method discussed in Section 3.2, with the opening price (F1) as the target variable. The output of the RFS feature selection method is the best subset of predictor features for each stock index, selected based on the lowest MAE. In the second stage, the output from the first stage is used as the input variables for the BiGRU-AM model to forecast the target variable, F1. Specifically, the data is processed through two layers of GRU in opposite directions, and the resulting data is then processed through a layer of AM as the final step.

### 2.5 Model Evaluation

The root mean square error (RMSE), mean absolute error (MAE), and the coefficient of determination, R-squared ($R^{2}$) were used to evaluate the accuracy of the output from the second stage, the forecasted opening price of the stock indices. The best model is chosen based on the lowest RMSE and MAE values, as well as the highest $R^{2}$. These metrics are computed as follows:


$$\text{RMSE}=\sqrt{\frac{1}{N}\sum {\mathrm{\backslash nolimits}}_{i=1}^{N}{\left(x_{i}-{\hat{x}}_{i}\right)}^{2}},$$

$$\text{MAE}=\frac{1}{N}\sum {\mathrm{\backslash nolimits}}_{i=1}^{N}\left|x_{i}-{\hat{x}}_{i}\right|,$$

$$R^{2}=\frac{\sum_{i=1}^{N}\left(x_{i}-{\bar{x}}_{i}\mathrm{\backslash rightleft}({\hat{x}}_{i}-{\hat{\bar{x}}}_{i}\right)}{\sqrt{\sum_{i=1}^{N}{\left(x_{i}-{\bar{x}}_{i}\right)}^{2}\sum_{i=1}^{N}{\left({\hat{x}}_{i}-{\hat{\bar{x}}}_{i}\right)}^{2}}}.$$

**where N is the total of data; $x_{i}$ is the actual opening price; ${\bar{x}}_{i}$ is the mean of the actual opening price and ${\hat{x}}_{i}$ is the forecasted opening price from the model.**

Table 3 presents the proposed model (M13) alongside twelve benchmark models. The benchmark models are categorized as follows:

**Table 3:** The proposed model and its benchmark models.

Model	Description
M1	Univariate GRU
M2	Univariate LSTM
M3	Univariate SVM
M4	Univariate MLP
M5	Multivariate GRU
M6	Multivariate LSTM
M7	Multivariate SVM
M8	Multivariate MLP
M9	Multivariate GRU with RFS feature selection
M10	Multivariate LSTM with RFS feature selection
M11	Multivariate SVM with RFS feature selection
M12	Multivariate MLP with RFS feature selection
M13 (Proposed)	Multivariate Bidirectional GRU with attention mechanism and RFS feature selection

- **M1–M4 (Univariate models):** These models use only the historical values of stock indices’ opening prices as the feature.
- **M5–M8 (Multivariate models):** These models use all available features for forecasting.
- **M9–M12 (RFS-selected features):** These models use features selected through the RFS (Random Feature Selection) method.

Each type of model—GRU, LSTM, SVM, and MLP—has three setups:

- **Zero predictors (Univariate):** M1 (GRU), M2 (LSTM), M3 (SVM), M4 (MLP)
- **All predictors (Multivariate):** M5 (GRU), M6 (LSTM), M7 (SVM), M8 (MLP)
- **RFS-selected predictors:** M9 (GRU), M10 (LSTM), M11 (SVM), M12 (MLP)

This structure allows for an easy and clear comparison of the impact of RFS feature selection on forecasting accuracy. Lastly, M13 model is:

- **M13 (Proposed model):** This model uses a two-stage approach with RFS feature selection in the first stage and a BiGRU-AM in the second stage.

Table 4 details the machine learning and deep learning hyperparameters for the benchmark models (M1–M12), while Table 5 details the deep learning hyperparameters for the proposed model (M13). The hyperparameters for each model were fine-tuned through trial and error. The specific process of hyperparameter selection is not discussed, as it is not the primary focus of this work.

**Table 4:** Hyperparameters used in the baseline models.

Model	Hyperparameter	Hyperparameter value
SVM	*kernel*	*rbf*
	*gamma*	*0.01*
	*epsilon*	*0.01*
	*shrinking*	*TRUE*
MLP	*hidden layer size*	*250*
	*solver*	*lbfgs*
	*activation*	*identity*
	*early stopping*	*TRUE*
GRU	*batch size*	*1024*
	*hidden layer size*	*250*
	*epoch*	*750*
	*solver*	*adam*
LSTM	*batch size*	*1024*
	*hidden layer size*	*250*
	*epoch*	*750*
	*solver*	*adam*

**Table 5:** Hyperparameters used in the RNN model in the proposed framework.

Model	Hyperparameter	Hyperparameter value
Bidirectional GRU	*batch size*	*1024*
	*hidden layer size*	*250*
	*epoch*	*750*
	*solver*	*adam*
	*initial learning rate*	*0.01*
	*decay steps*	*200*
	*decay rate*	*0.2*
Attention Mechanism	*weight initializer*	*He normal* [44]
	*bias initializer*	*Zeros*

## 3. Results and discussion

The performance of the proposed model (M13) is assessed and compared with twelve benchmark models (M1 – M12) various metrics, including RMSE, MAE, and R2 values. Additionally, visual representations, such as plots of actual opening prices and forecasted values from all thirteen models for all ten stock indices, are provided to illustrate the findings and underscore the effectiveness of the proposed two-stage approach in predicting the opening prices of stock indices.

### 3.1 Results of the feature selection

Table 6 displays the feature selection results. F2 (Highest prices), F3 (Lowest prices), F6 (Change), and F7 (Price change ratio) are the four most important features for predicting the opening price. Except for the FTSE 100 and IPC, F2 and F3 are selected for all the indices. F6 is selected for all indices except CAC 40 and AEX, while F7 is only excluded in FTSE 100 and BEL 20. Notably, F8 (amplitude) is not selected in any model for any stock.

**Table 6:** Selected features using the RFS feature selection method for the stock indices investigated.

Stock Indices	Features selected	Description
Dow Jones	F2, F3, F4, F6, F7	High, Low, Close, Change, Price change ratio
Nasdaq	F2, F3, F6, F7	High, Low, Change, Price change ratio
Nikkei 225	F2, F3, F6, F7	High, Low, Change, Price change ratio
FTSE 100	F4, F5, F6	Close, Volume, Change
S&P 500	F2, F3, F4, F6, F7	High, Low, Close, Change, Price change ratio
CAC 40	F2, F3, F4, F7	High, Low, Close, Price change ratio
IPC	F4, F6, F7	Close, Volume, Price change ratio
DAX	F2, F3, F6, F7	High, Low, Change, Price change ratio
AEX	F2, F3, F7	High, Low, Price change ratio
BEL 20	F2, F3, F6	High, Low, Change

The highest price (F2) and the lowest price (F3) are critical features in predicting the opening price of stock indices because they reflect the trading range and provide insights into market volatility. The opening price of a stock reflects adjustments based on new information and captures the market sentiment and volatility from the previous trading day [45]. The highest and lowest prices show the range within which a stock has traded, indicating its potential volatility. This information is particularly valuable for day-traders and financial analysts, as it helps them gauge market sentiment and predict future opening prices. By incorporating the highest and lowest prices into predictive models, traders can make more informed decisions, aiming to maximize profit and minimize risk.

The change (F6) represents the price changes from its previous value and the price change ratio (F7) normalizes these changes, enabling direct comparison across different stocks and indices. It is often utilized by portfolio managers and investors to evaluate performance and risk across various asset classes. This normalization is crucial for making comparisons and evaluating performance across different stocks and indices [46]. Additionally, change is associated with volatility, underscoring the importance of considering volatility levels in predictive models [47]. The selection of these two features highlights their significance in capturing market dynamics and aiding in accurate forecasting.

Amplitude (F8) was not selected as a predictor feature by any model in predicting the opening price. Amplitude reflects the degree of stock activity [42]. In this study, the importance of features was examined in groups of subsets. This indicates that while amplitude could be an important feature on its own, its dependencies on other features in predicting the opening price might not be significant. The proposed RFS feature selection has been designed to focus on the importance of the features, while considering their interactions with other features, hence producing more reliable and accurate forecasts.

### 3.2. Forecasting accuracy evaluation

The RMSE, MAE, and $R^{2}$ values of the proposed model and its benchmark models for the selected dataset are shown in Table 7. M13 (the proposed model) produces the lowest RMSE and MAE values for almost all ten indices, except for the MAE of FTSE 100 and DAX. For these two cases, the proposed model’s MAE values are close to and comparable to the lowest values produced by M10 for the FTSE 100 and M9 for the DAX. For all indices, the $R^{2}$ values produced by the M13 model are the highest. The proposed model outperforms the other twelve benchmark models.

**Table 7:** The forecasting performance of different models on 10 stock indices.

Model	Dow Jones				NASDAQ				Nikkei 225			
RMSE		MAE	$R^{2}$	RMSE		MAE	$R^{2}$	RMSE	MAE		$R^{2}$
M1	777.602		476.781	0.965	364.953		254.592	0.977	526.512	380.086		0.976
M2	856.231		662.977	0.957	449.139		319.110	0.966	507.300	367.612		0.988
M3	505.340		350.375	0.985	250.710		195.152	0.989	367.612	272.546		0.988
M4	612.518		428.874	0.978	351.166		273.239	0.979	355.489	269.976		0.989
M5	271.913		245.634	0.996	299.258		278.977	0.985	291.419	277.169		0.993
M6	442.666		417.246	0.989	249.703		230.508	0.989	250.198	231.457		0.995
M7	709.376		654.807	0.971	560.209		519.785	0.947	308.257	265.522		0.992
M8	661.014		552.255	0.975	415.376		333.204	0.971	313.175	250.155		0.991
M9	225.070		200.887	0.997	132.807		110.705	0.997	273.397	255.553		0.994
M10	299.990		274.775	0.995	167.457		149.493	0.995	194.447	175.528		0.997
M11	639.365		581.179	0.976	547.935		513.730	0.949	300.205	262.550		0.992
M12	339.015		281.038	0.993	196.686		160.270	0.993	217.028	185.736		0.996
**M13 (Proposed)**	**196.539**		**157.489**	**0.998**	**84.103**		**65.548**	**0.999**	**112.952**	**94.329**		**0.999**
Model	FTSE 100				S&P 500				CAC 40			
	RMSE		MAE	$R^{2}$	RMSE		MAE	$R^{2}$	RMSE	MAE		$R^{2}$
M1	147.286		86.174	0.942	88.799		59.135	0.979	123.629	87.797		0.979
M2	149.076		87.441	0.941	90.814		59.090	0.978	94.926	64.843		0.988
M3	106.004		66.053	0.970	59.961		45.423	0.991	96.720	66.987		0.987
M4	128.206		84.649	0.956	90.128		64.768	0.979	97.245	70.210		0.987
M5	47.379		39.011	0.994	16.243		13.455	0.999	71.929	67.129		0.993
M6	56.034		49.607	0.992	44.673		41.780	0.995	80.612	76.217		0.991
M7	66.914		56.521	0.988	128.594		119.728	0.957	67.213	62.505		0.994
M8	67.479		59.473	0.988	65.544		50.820	0.988	74.812	67.620		0.992
M9	23.421		22.642	0.999	19.578*		16.552*	0.999*	87.050*	82.872*		0.990*
M10	15.496		**10.753**	0.999	24.702		21.756	0.998	68.657	63.640		0.994
M11	31.726		26.328	0.997	117.121		107.726	0.964	70.977	66.391		0.993
M12	39.287		12.831	0.996	45.447		35.260	0.995	72.019	65.077		0.993
**M13 (Proposed)**	**13.278**		12.512	**1.000**	**17.770**		**14.150**	**0.999**	**66.823**	**61.186**		**0.994**
Model	IPC			DAX			AEX			BEL 20		
	RMSE	MAE	$R^{2}$	RMSE	MAE	$R^{2}$	RMSE	MAE	$R^{2}$	RMSE	MAE	$R^{2}$
M1	1169.17	839.480	0.963	389.811	251.265	0.949	28.909	24.231	0.919	69.602	44.867	0.974
M2	1368.06	1099.44	0.949	379.259	244.499	0.952	13.183	9.032	0.983	71.784	50.248	0.972
M3	691.505	520.787	0.987	248.446	168.559	0.979	10.096	7.301	0.990	181.827	138.249	0.821
M4	991.321	764.870	0.973	408.590	277.278	0.944	12.423	9.006	0.985	85.589	57.089	0.960
M5	752.697	114.301	0.999	80.800	60.258	0.998	5.025	4.283	0.998	27.140	22.588	0.996
M6	188.280	154.575	0.999	78.199	57.905	0.998	3.054	2.181	0.999	33.877	30.100	0.994
M7	210.657	168.188	0.999	157.142	135.550	0.992	3.474	0.615	0.999	93.246	79.125	0.953
M8	611.847	469.180	0.990	200.849	149.882	0.986	6.858	5.491	0.995	38.770	31.958	0.992
M9	234.039	190.212	0.999	65.146	**44.813**	0.999	3.299	2.602	0.999	28.443*	24.953*	0.996*
M10	179.346	124.062	0.999	68.284	51.004	0.998	3.753	3.066	0.999	35.867*	33.026*	0.993*
M11	150.692	102.467	0.999	157.237*	138.469*	0.992*	3.310	2.509	0.999	88.918	72.382	0.957
M12	251.825	192.585	0.998	75.530	53.833	0.998	4.436	3.757	0.998	34.761	29.496	0.993
**M13 (Proposed)**	**170.239**	**108.053**	**0.999**	**64.206**	44.970	**0.999**	**2.990**	**2.214**	**0.999**	**25.485**	**21.316**	**0.996**

Note: Values in **bold** indicate the lowest RMSE and MAE, and the highest $R^{2}$ values among all the models. Values marked with asterisk * indicate models for which the RFS feature selection do not show improvement.

According to the results of Table 7, models with all the predictor features (M5 – M9) outperform models with zero predictor features (M1 – M4) in all cases. However, they are outperformed by their counterpart models with RFS-selected features (M10 – M12) in almost all cases, except for five instances: the GRU model for S&P 500, CAC 40, and BEL 20, the SVM model for DAX, and the LSTM model for BEL 20. This suggests that predictor features in multivariate model yields important temporal data, resulting in better model prediction when compared to their counterpart univariate models. Furthermore, the findings show that RFS feature selection efficiently reduce the dimensions of the data by selecting only the best subset of features that are essential in predicting the opening price which eventually prevent the overfitting problem. This is why models with RFS-selected features (M10 – M12) forecast perform better than the models with all features present (M5 – M9).

In addition, the proposed two-stage model (M13) provides the most accurate predictions across all cases. Forecasts from M13 produces the lowest RMSE and MAE, and highest $R^{2}$ in all but two cases: MAE in FTSE 100 and DAX. Even so, the MAE values for M13 in these two cases are close and comparable to the lowest MAE by M10 for FTSE 100 and by M9 for DAX. Following the selection in the first stage through RFS feature selection, the BiGRU-AM model was used for multivariate forecasting. SVM, MLP, GRU, and LSTM are the four baseline for the benchmark models used in this study. SVM and MLP are supervised learning techniques that were originally proposed as classifier algorithms before being extended to regression problems. GRU and LSTM, on the other hand, are two RNN examples [48]. RNN is well-suited for stock price forecasts due to its ability to handle sequential data and capture temporal dependencies effectively [49]. Due to its inherent advantages, GRU was selected as the foundational model to be to be extended. In [50], GRU was demonstrated to have shorter training time compared to LSTM. This is because GRU has fewer gates than LSTM and thus requires fewer parameters to train the data. As a result, it takes less time for the model to converge, overcoming the vanishing gradient problem. As highlighted in Section 1, the bidirectional version of GRU was used in this study due to its advantage over the unidirectional counterpart, as it helps avoid the problem of information loss that can occur in a unidirectional system [51]. Furthermore, the addition of AM to the model aids in assigning higher weights to critical temporal information that would otherwise be missed, improving forecast accuracy even further.

**Fig 4:**
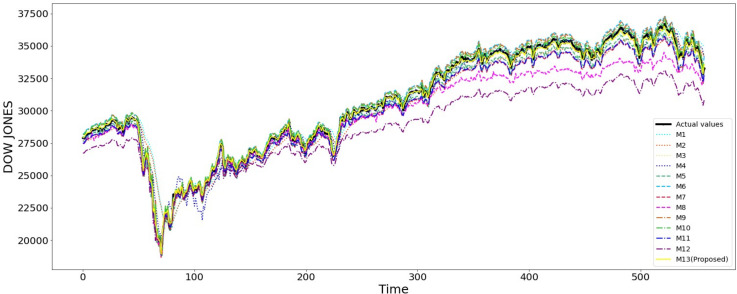
The actual Dow Jones opening price and its forecasted values from different models.

**Fig 5:**
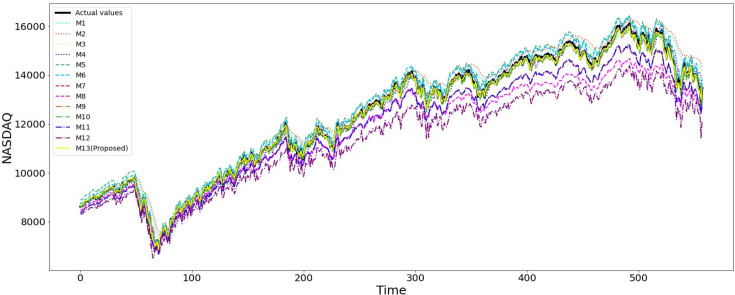
The actual Nasdaq opening price and its forecasted values from different models.

Overall, the plots of actual versus predicted opening prices for all ten stock indices suggest that the proposed model (M13) provides the most accurate predictions. Figs 4 and 5 display the plots for the Dow Jones and Nasdaq, respectively, while the plots for the other eight indices are available in the S1 File. In these plots, the black line represents the actual values, and the yellow line represents the predicted values from the proposed model (M13). These figures help to visualize how closely the proposed model (M13) follows the actual market fluctuations and captures the peaks and troughs of the indices, particularly for the Dow Jones, Nasdaq, and S&P 500. This observation aligns with and supports the statistical measures in Table 7, which indicate that the proposed two-stage model M13 outperforms the other twelve benchmark models in predicting opening prices of stock indices. By accurately capturing the trends in the stock indices, the proposed model confirms its superiority in model performance, highlighting the effectiveness of the methodology presented in this study.

## 4. Conclusions, limitations, and future directions

A two-stage model for forecasting financial time series was introduced in this study. In the first step, an RF subset-based feature selection method is used to choose the best subset of features by looking at all subsets of features using the repeated k-fold cross validation method. This is done to ensure that the forecasting model does not incorporate features that are redundant. In the second stage, the selected features are fitted as inputs to a bidirectional GRU model with AM. The Bidirectional GRU model considers both forward and backward temporal aspects of the data, thereby enhancing model accuracy. Additionally, AM contributes by emphasizing critical temporal information. By a wide margin, this combination outperformed the other benchmark models discussed in this paper. Using the suggested RF subset-based approach to choose features helped find the best predictors to improve the performance of the bidirectional GRU model with AM.

The combination of BiGRU, attention mechanisms, and feature selection techniques significantly improves the accuracy and efficiency of stock price prediction models. These methods address the inherent complexities of stock data, such as nonlinearity and temporal dependencies, leading to more precise and interpretable predictions [52,53]. This work also has practical implications in that investors can use it to make investment decisions. This is because a forecasting model with higher prediction accuracy of stock price trends will aid in lowering the risk of profitably investing capital and maximizing investment profit.

It would be interesting to extend the current feature selection approach by adding a feature filtration layer based on causality tests to remove redundant or weakly correlated features before model training. In this way, the variables that provide the highest information to the model are selected, hence, it could reduce computational complexity while maintaining the accuracy, making the model more suitable for real-time stock prediction. The computational time for the feature selection method can also be further enhanced especially for larger datasets. Prior studies have demonstrated that causality-based feature selection improve the efficiency of deep learning model in financial forecasting [54]. To refine the prediction error and improve prediction accuracy, an error-corrected version of the bidirectional GRU with AM model is also of interest. Error correction models are proven to significantly improve the performance of BiGRU models by enhancing prediction accuracy, handling nonlinear data characteristics, reducing overfitting, and increasing model efficiency [55,56].

## Supporting information

S1 File S1 Fig. The actual Nikkei 225 opening price and its forecasted values from different models. S2 Fig. The actual FTSE 100 opening price and its forecasted values from different models. S3 Fig. The actual S&P 500 opening price and its forecasted values from different models. S4 Fig. The actual CAC 40 opening price and its forecasted values from different models. S5 Fig. The actual IPC opening price and its forecasted values from different models. S6 Fig. The actual DAX opening price and its forecasted values from different models. S7 Fig. The actual AEX opening price and its forecasted values from different models. S8 Fig. The actual BEL 20 opening price and its forecasted values from different models.
(ZIP)
