# Correction: A two-stage forecasting model using random forest subset-based feature selection and BiGRU with attention mechanism: Application to stock indices

**DOI:** 10.1371/journal.pone.0345034

**Published:** 2026-03-13

**Authors:** Shafiqah Azman, Dharini Pathmanathan, Vimala Balakrishnan

In the Model Evaluation subsection of the Methodology, the equation presented for the coefficient of determination$R^{2}$is incorrect. Please view the complete, correct equation here:


$$\begin{array}{c} R^{\text{2}}=\text{1}-\frac{\sum_{i=\text{1}}^{N}{\left(x_{i}-{\hat{x}}_{i}\right)}^{\text{2}}}{\sum_{i=\text{1}}^{N}{\left(x_{i}-\overset{―}{x}\right)}^{\text{2}}}\; \end{array}$$

## References

[1] Azman S, Pathmanathan D, Balakrishnan V. A two-stage forecasting model using random forest subset-based feature selection and BiGRU with attention mechanism: Application to stock indices. PLoS One. 2025;20(5):e0323015. doi: 10.1371/journal.pone.0323015 40344172 PMC12064028

