# Supplementary figures and images for: A two-stage forecasting model using random forest subset-based feature selection and BiGRU with attention mechanism: Application to stock indices
Source: PLoS One. 2025 May 9;20(5):e0323015. doi: 10.1371/journal.pone.0323015 (PMC12064028; doi:10.1371/journal.pone.0323015)

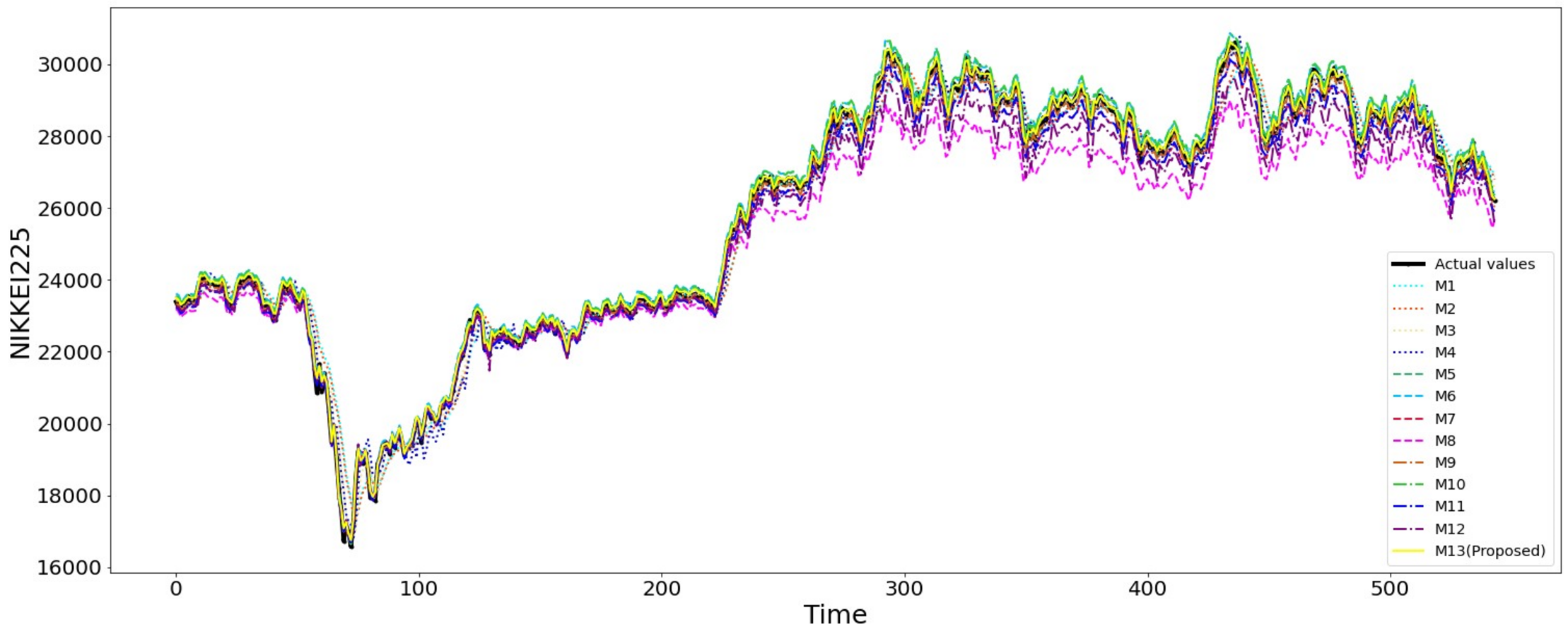

Supplement: S1 File — S2 Fig. The actual FTSE 100 opening price and its forecasted values from different models. S3 Fig. The actual S&P 500 opening price and its forecasted values from different models. S4 Fig. The actual CAC 40 opening price and its forecasted values from different models. S5 Fig. The actual IPC opening price and its forecasted values from different models. S6 Fig. The actual DAX opening price and its forecasted values from different models. S7 Fig. The actual AEX opening price and its forecasted values from different models. S8 Fig. The actual BEL 20 opening price and its forecasted values from different models. (ZIP) [file pone.0323015.s001.zip › S1 Fig.tif]

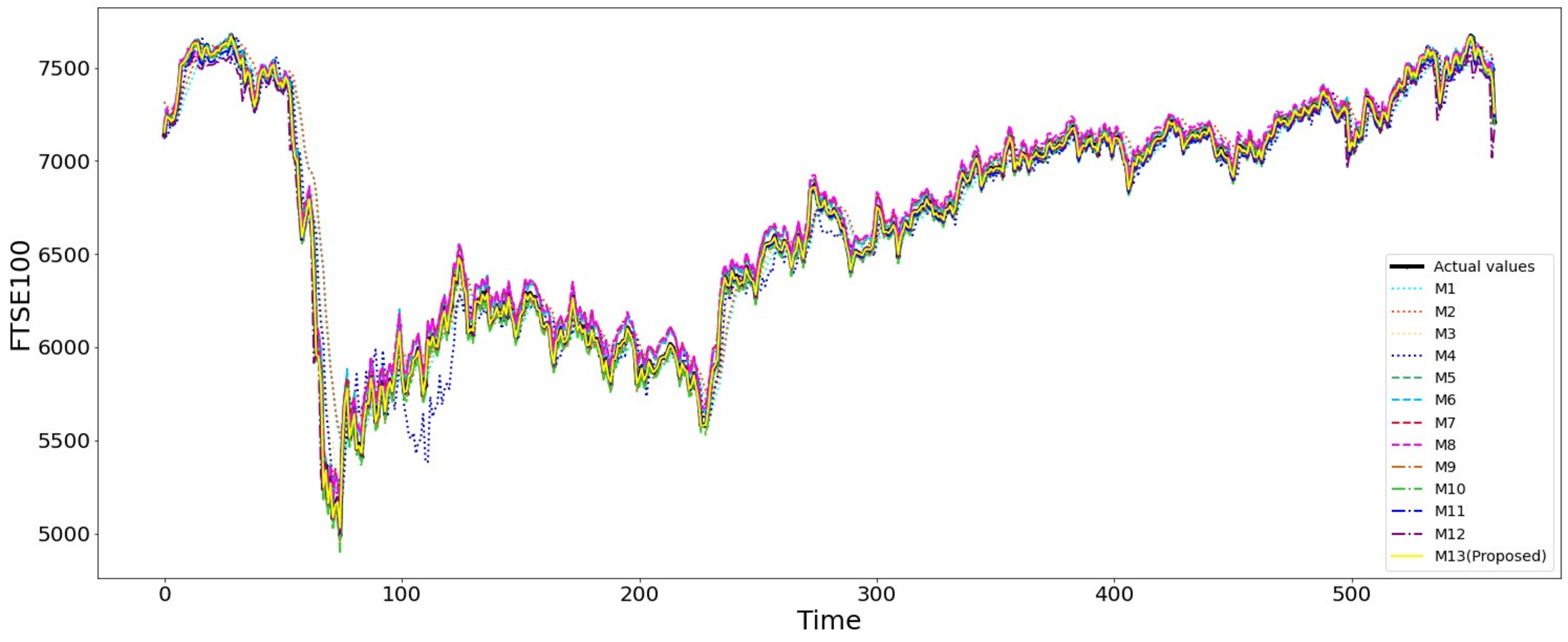

Supplement: S1 File — S2 Fig. The actual FTSE 100 opening price and its forecasted values from different models. S3 Fig. The actual S&P 500 opening price and its forecasted values from different models. S4 Fig. The actual CAC 40 opening price and its forecasted values from different models. S5 Fig. The actual IPC opening price and its forecasted values from different models. S6 Fig. The actual DAX opening price and its forecasted values from different models. S7 Fig. The actual AEX opening price and its forecasted values from different models. S8 Fig. The actual BEL 20 opening price and its forecasted values from different models. (ZIP) [file pone.0323015.s001.zip › S2 Fig.tif]

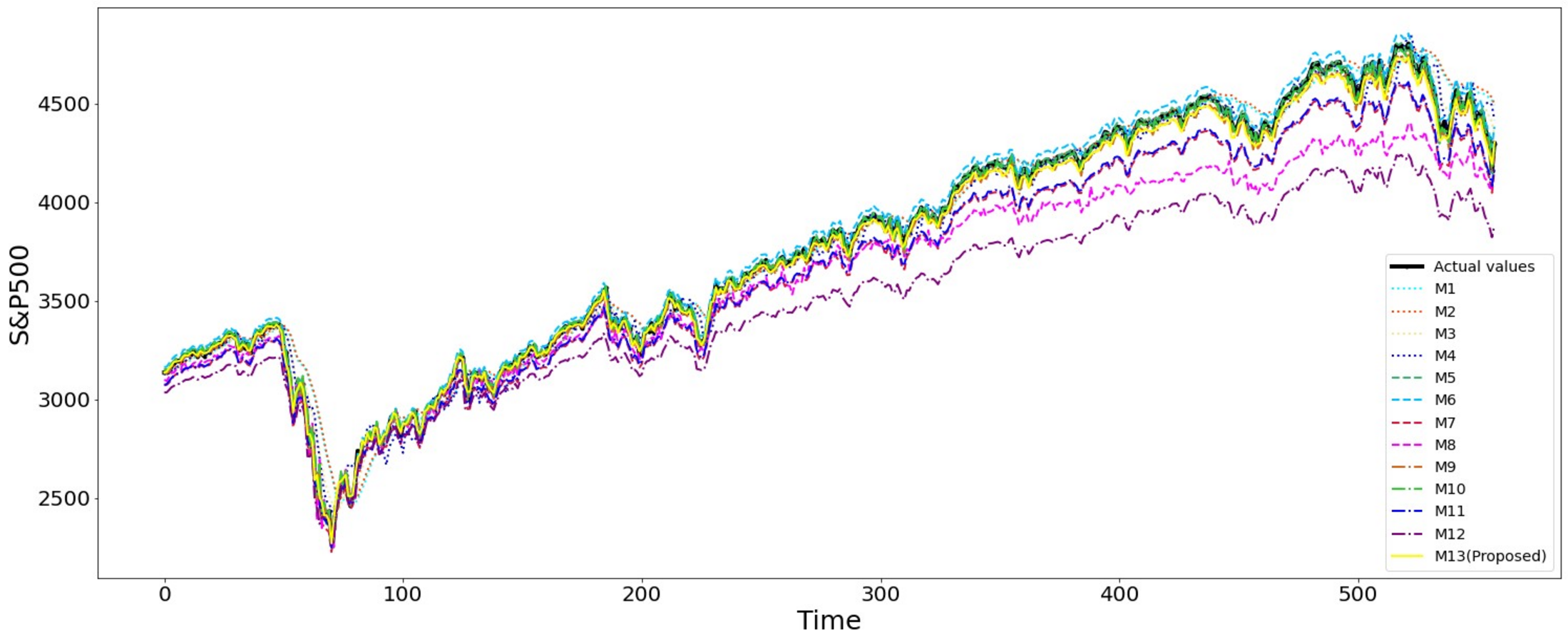

Supplement: S1 File — S2 Fig. The actual FTSE 100 opening price and its forecasted values from different models. S3 Fig. The actual S&P 500 opening price and its forecasted values from different models. S4 Fig. The actual CAC 40 opening price and its forecasted values from different models. S5 Fig. The actual IPC opening price and its forecasted values from different models. S6 Fig. The actual DAX opening price and its forecasted values from different models. S7 Fig. The actual AEX opening price and its forecasted values from different models. S8 Fig. The actual BEL 20 opening price and its forecasted values from different models. (ZIP) [file pone.0323015.s001.zip › S3 Fig.tif]

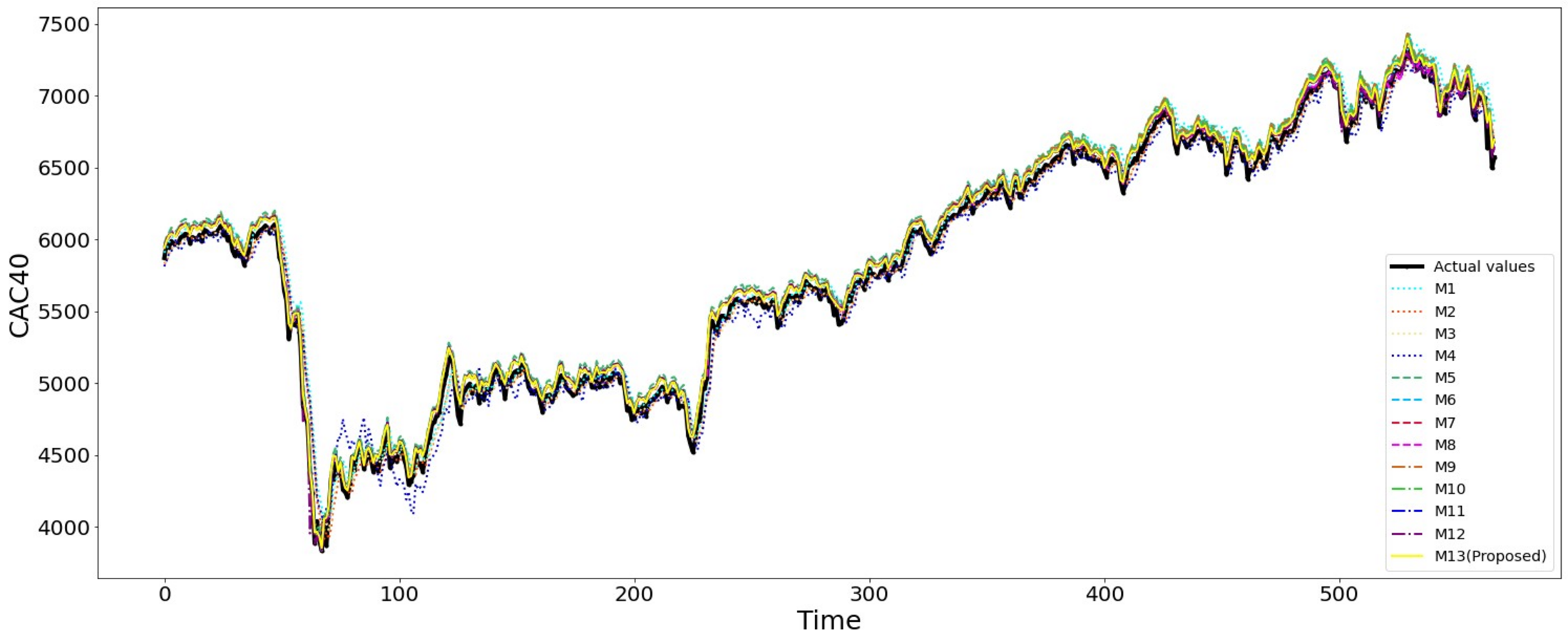

Supplement: S1 File — S2 Fig. The actual FTSE 100 opening price and its forecasted values from different models. S3 Fig. The actual S&P 500 opening price and its forecasted values from different models. S4 Fig. The actual CAC 40 opening price and its forecasted values from different models. S5 Fig. The actual IPC opening price and its forecasted values from different models. S6 Fig. The actual DAX opening price and its forecasted values from different models. S7 Fig. The actual AEX opening price and its forecasted values from different models. S8 Fig. The actual BEL 20 opening price and its forecasted values from different models. (ZIP) [file pone.0323015.s001.zip › S4 Fig.tif]

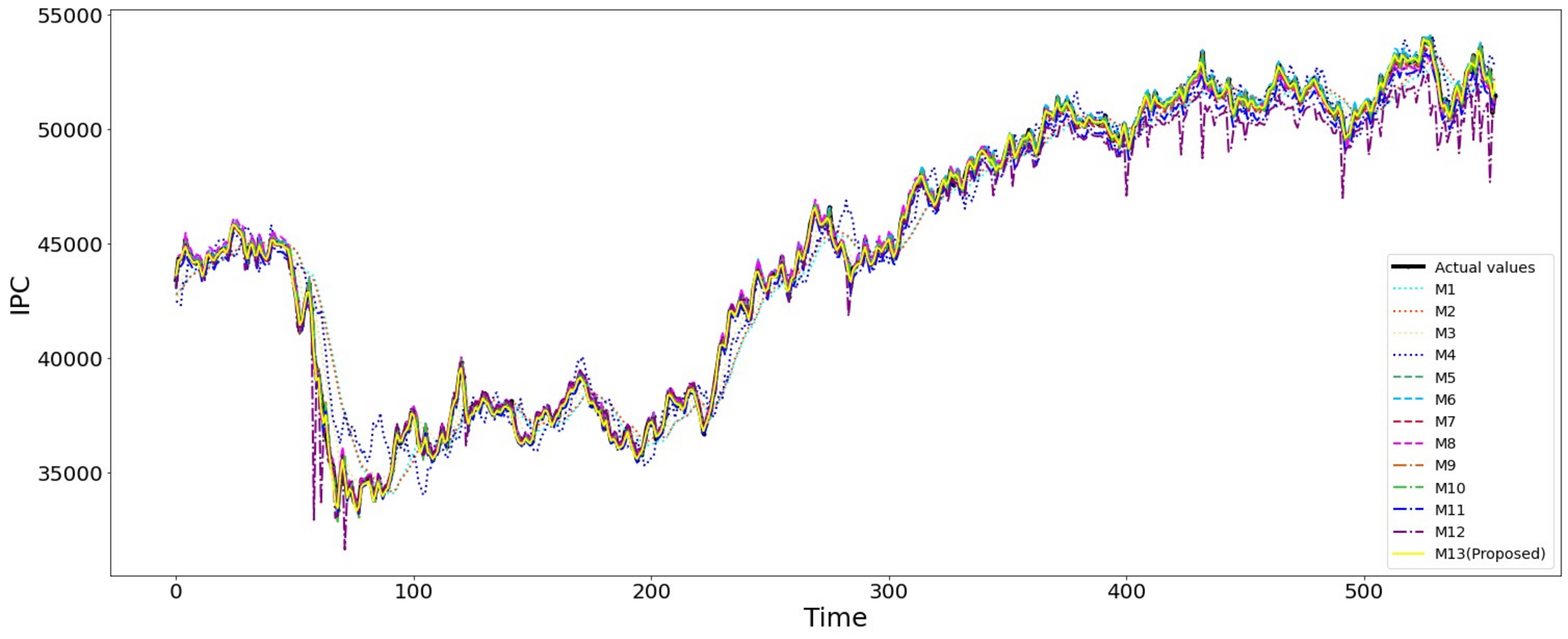

Supplement: S1 File — S2 Fig. The actual FTSE 100 opening price and its forecasted values from different models. S3 Fig. The actual S&P 500 opening price and its forecasted values from different models. S4 Fig. The actual CAC 40 opening price and its forecasted values from different models. S5 Fig. The actual IPC opening price and its forecasted values from different models. S6 Fig. The actual DAX opening price and its forecasted values from different models. S7 Fig. The actual AEX opening price and its forecasted values from different models. S8 Fig. The actual BEL 20 opening price and its forecasted values from different models. (ZIP) [file pone.0323015.s001.zip › S5 Fig.tif]

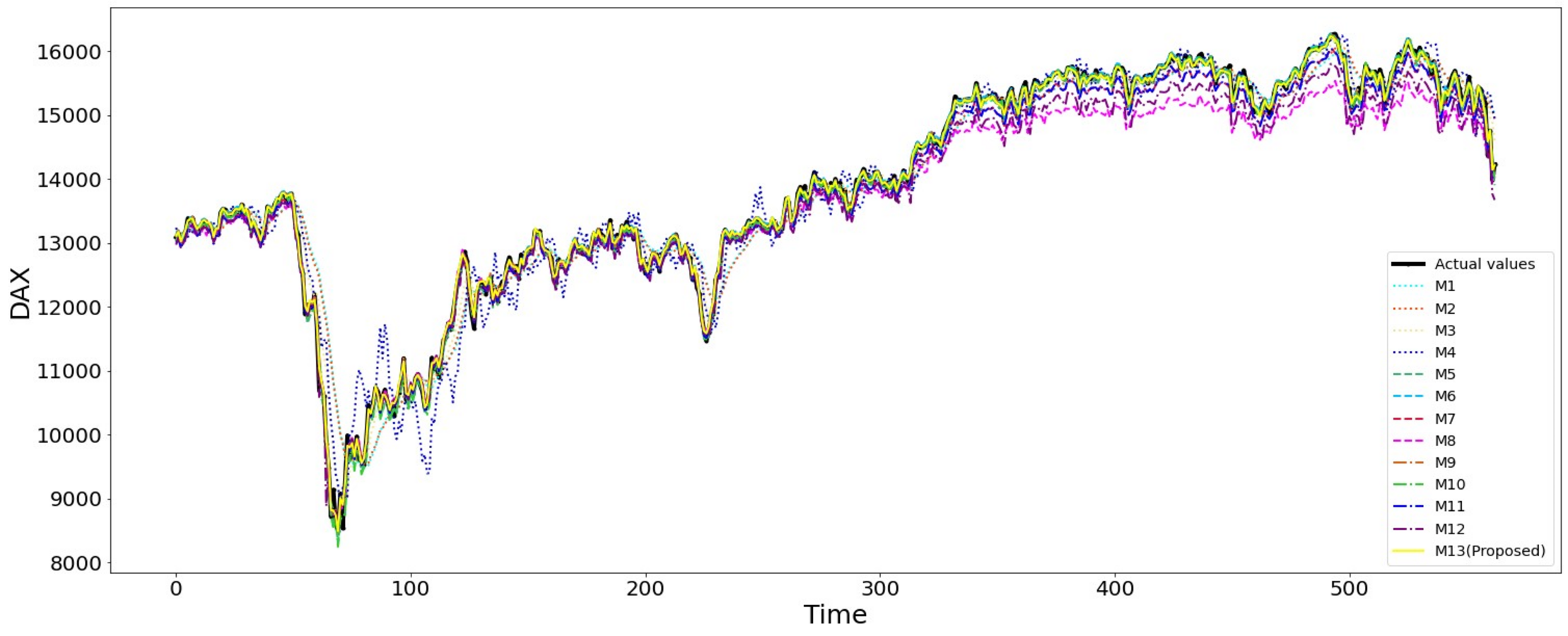

Supplement: S1 File — S2 Fig. The actual FTSE 100 opening price and its forecasted values from different models. S3 Fig. The actual S&P 500 opening price and its forecasted values from different models. S4 Fig. The actual CAC 40 opening price and its forecasted values from different models. S5 Fig. The actual IPC opening price and its forecasted values from different models. S6 Fig. The actual DAX opening price and its forecasted values from different models. S7 Fig. The actual AEX opening price and its forecasted values from different models. S8 Fig. The actual BEL 20 opening price and its forecasted values from different models. (ZIP) [file pone.0323015.s001.zip › S6 Fig.tif]

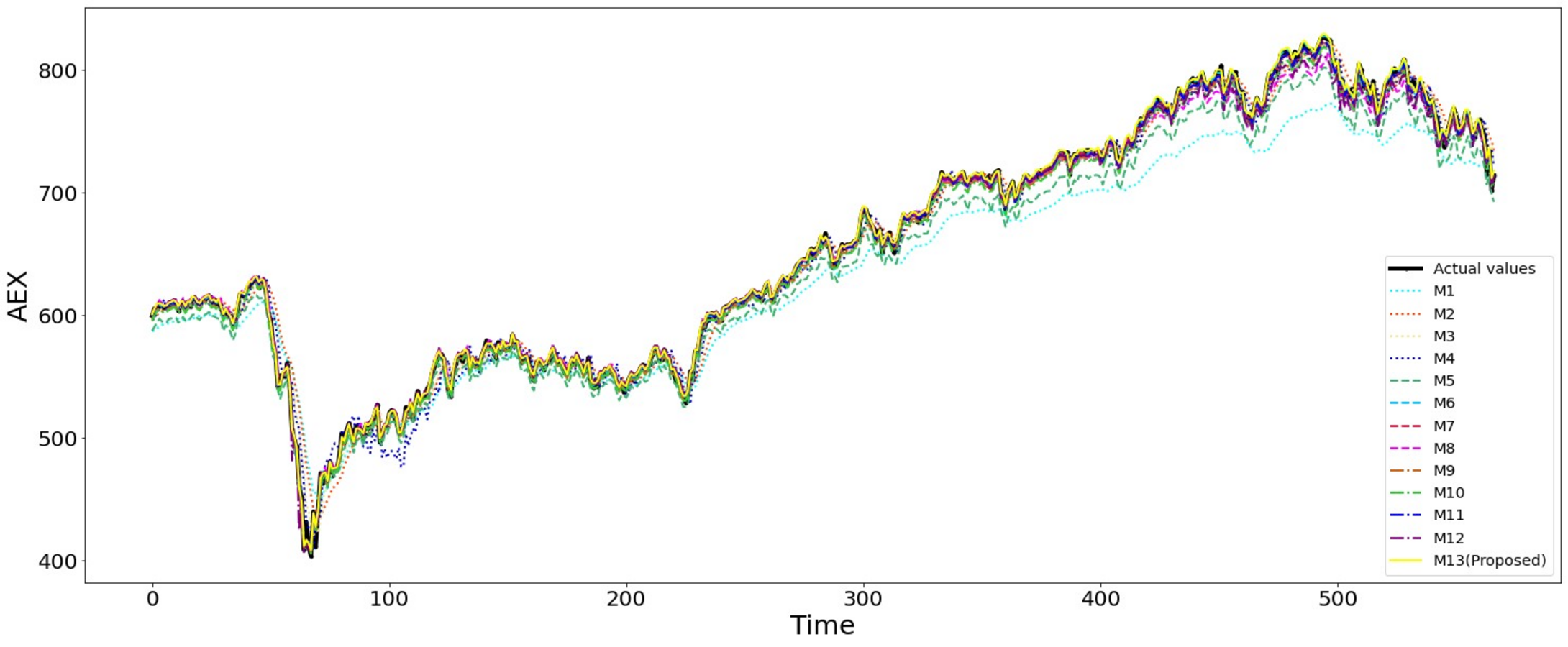

Supplement: S1 File — S2 Fig. The actual FTSE 100 opening price and its forecasted values from different models. S3 Fig. The actual S&P 500 opening price and its forecasted values from different models. S4 Fig. The actual CAC 40 opening price and its forecasted values from different models. S5 Fig. The actual IPC opening price and its forecasted values from different models. S6 Fig. The actual DAX opening price and its forecasted values from different models. S7 Fig. The actual AEX opening price and its forecasted values from different models. S8 Fig. The actual BEL 20 opening price and its forecasted values from different models. (ZIP) [file pone.0323015.s001.zip › S7 Fig.tif]

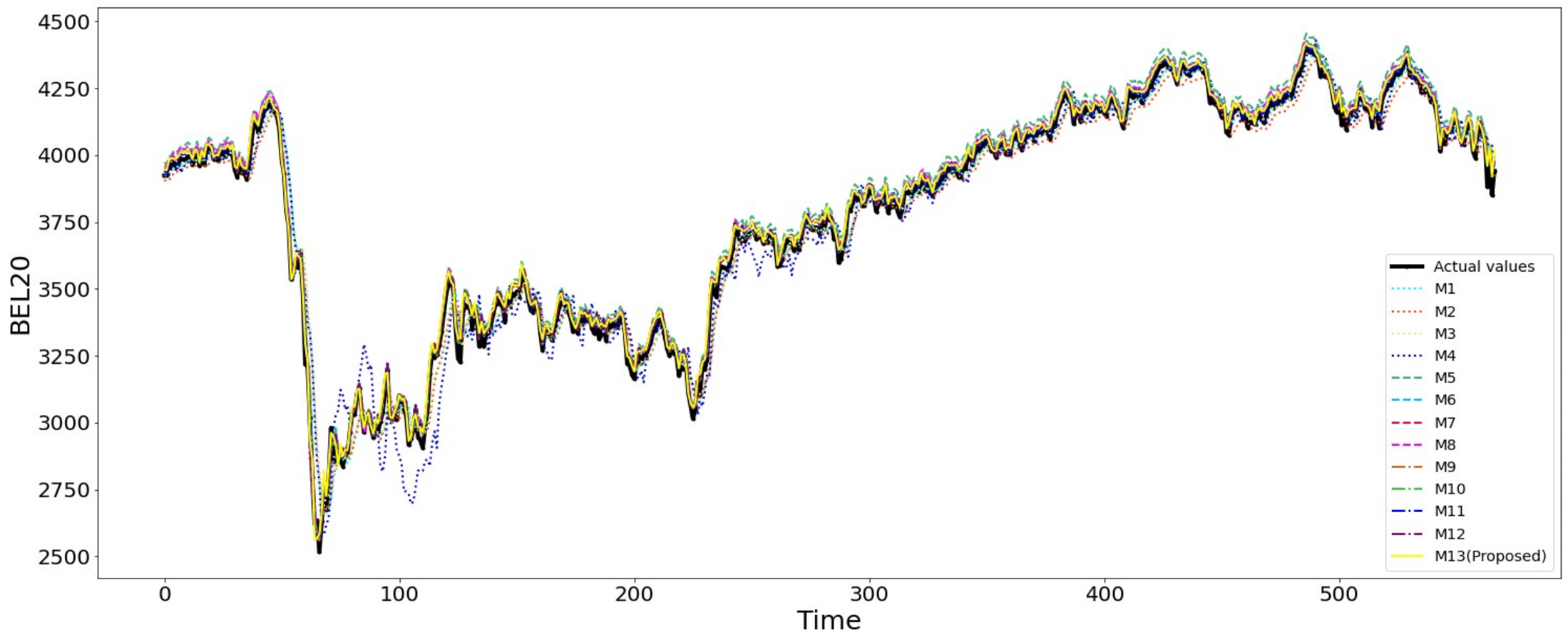

Supplement: S1 File — S2 Fig. The actual FTSE 100 opening price and its forecasted values from different models. S3 Fig. The actual S&P 500 opening price and its forecasted values from different models. S4 Fig. The actual CAC 40 opening price and its forecasted values from different models. S5 Fig. The actual IPC opening price and its forecasted values from different models. S6 Fig. The actual DAX opening price and its forecasted values from different models. S7 Fig. The actual AEX opening price and its forecasted values from different models. S8 Fig. The actual BEL 20 opening price and its forecasted values from different models. (ZIP) [file pone.0323015.s001.zip › S8 Fig.tif]
